# deltaHED predicts survival and immune evasion in PD‐1 blockade therapy: A multi‐cohort study across three cancer types

**DOI:** 10.1002/ctm2.70595

**Published:** 2026-01-28

**Authors:** Jianying Xu, Xiaoli Wei, Jicheng Yao, Ujjwal Mukund Mahajan, Ulf Dietrich Kahlert, Run Shi, Kaiying Zhang, Ahmed Alnatsha, Zhengyi Qian, Fei Han, Fenghua Wang

**Affiliations:** ^1^ Department of Medical Oncology Sun Yat‐sen University Cancer Center, State Key Laboratory of Oncology in South China, Collaborative Innovation Center for Cancer Medicine Guangzhou China; ^2^ Department of Medicine II LMU University Hospital Munich Germany; ^3^ Research and Development Department Shanghai OrigniMed Co., Ltd Shanghai China; ^4^ Department of Pharmacology and Toxicology National Institute of Pharmaceutical Education and Research (NIPER) Mohali India; ^5^ Department of Molecular and Experimental Surgery, Clinic for General, Visceral, Vascular and Transplantation Surgery Medical Faculty and University Hospital Magdeburg, Otto‐von‐Guericke University Magdeburg Germany; ^6^ Department of Oncology The First Affiliated Hospital of Nanjing Medical University Nanjing China; ^7^ Departments of Radiation Oncology Sun Yat‐sen University Cancer Center, State Key Laboratory of Oncology in South China, Collaborative Innovation Center for Cancer Medicine Guangzhou China

**Keywords:** antigen presentation, anti‐programmed death‐1 immunotherapy, esophageal squamous cell carcinoma, human leukocyte antigen class I, nasopharyngeal carcinoma

## Abstract

The prognostic relevance of HLA class I (HLA‐I)‐mediated immunity in cancer immunotherapy remains unclear. We introduce deltaHED, a novel metric that quantifies evolutionary divergence between germline and tumour‐acquired HLA‐I alleles, integrating both inherited and somatic immunogenetic variation. Using whole‐exome sequencing, we analysed deltaHED across three independent cohorts: 164 patients with recurrent/metastatic nasopharyngeal carcinoma (RM/NPC) from the POLARIS‐02 trial (PD‐1 monotherapy), 88 melanoma patients receiving PD‐1 monotherapy, and 477 esophageal squamous cell carcinoma (ESCC) patients from the JUPITER‐06 trial (PD‐1 plus chemotherapy vs. chemotherapy alone). High deltaHED was significantly associated with increased tumour mutational burden and neoantigen load (*p* < .001), but predicted worse progression‐free survival (PFS) and overall survival (OS) in patients receiving PD‐1 blockade across all three cancers. In ESCC, this association was observed only in the immunotherapy arm, not in patients treated with chemotherapy alone. High deltaHED also correlated with increased mutations in antigen‐processing and T‐cell receptor pathways. These findings establish deltaHED as a clinically relevant biomarker of immune divergence with potential to improve patient stratification and guide personalised immunotherapy strategies.

## Graphical abstract

deltaHED predicts survival and immune evasion in PD‐1 blockade therapy: a multi‐cohort study across three cancer types. deltaHED quantifies germlinesomatic HLA‐I divergence and predicts survival and immune evasion in PD‐1 blockade therapy. Across nasopharyngeal carcinoma, melanoma, and esophageal squamous cell carcinoma, high deltaHED is associated with poor prognosis and increased mutations affecting antigen presentation and T‐cell activation. (A) Germline HLA evolutionary divergence (HED) reflects the inherited diversity of human leukocyte antigen (HLA) alleles (e.g., HLA‐A:01:01, HLA‐A:02:01, HLA‐B:07:02, HLA‐B:08:01, HLA‐C:03:03, HLA‐C:07:01), enabling the presentation of diverse immunogenic antigens (different colour in ball) and potential of broad T‐cell activation. (B) Somatic HED: tumour cells undergo loss of heterozygosity (LOH), leading to the loss of specific HLA alleles (e.g., HLA‐A:01:01 LOH, HLA‐B :08:01 LOH, HLA‐C:07:01 LOH) and reduced antigen presentation, resulting in immune evasion. (C) deltaHED (quantifying HLA diversity loss): deltaHED is calculated as the difference between germline HED and somatic HED, reflecting the extent of tumour‐mediated HLA diversity loss. High deltaHED is associated with higher mutation percentage of antigen presentation pathway and poor survival in patients receiving PD‐1 blockade therapy (validated in nasopharyngeal carcinoma [NPC], melanoma, and esophageal squamous cell carcinoma [ESCC] cohorts). HLA alleles: red (HLA‐A), blue (HLA‐B), green (HLA‐C). T cells: green (activated), grey (inactivated). The abstract graph is created in https://BioRender.com.

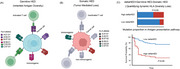

## 1 INTRODUCTION

Blockade of programmed cell death protein 1 (PD‐1) has transformed the therapeutic landscape for advanced malignancies. However, only a fraction of patients achieve long‐term benefit.^1^, ^2^, ^3^, ^4^, ^5^ Although tumour mutational burden (TMB) and PD‐L1 expression have been extensively investigated, their ability to consistently predict response varies among tumour types. It underscores the need for novel biomarkers that capture fundamental mechanisms of immune evasion.^6^, ^7^

HLA class I molecules play a pivotal role in antitumour effect by presenting tumour‐derived peptides to cytotoxic CD8+ T lymphocytes.^8^ The diversity of HLA‐I alleles—determined by germline genetics, dictates the breadth of antigen presentation.^9^ Recent investigations have proposed HLA evolutionary divergence (HED) as an indicator of functional diversity among HLA‐I alleles.^10^ HED measures the evolutionary distance between HLA‐I alleles within an individual, with higher HED values reflecting alleles that diverged earlier in evolution and thus recognise more distinct antigenic repertoires. Germline HED, therefore, represents the inherent potential of an individual's HLA‐I system to present diverse neoantigens.

Emerging evidence suggests that germline HED can influence the tumour immune microenvironment (TIME) and the efficacy of PD‐1 blockade.^11^, ^12^ Individuals with higher germline HED exhibit broader peptide presentation, facilitating efficient priming and expansion of cytotoxic T cells, thereby fostering a more inflamed and immune‐active TIME. Conversely, low germline HED restricts antigen presentation diversity, contributing to immune exclusion and tolerance. Several studies have linked high HED to improved clinical outcomes under PD‐1 blockade in gastrointestinal^13^ and non‐small cell lung cancers.^14^ However, other investigations have reported no predictive value of germline HLA profiles in pan‐cancer cohorts,^15^ suggesting that germline HED alone cannot fully explain patient heterogeneity in immunotherapy response. This discrepancy implies that tumour intrinsic alterations may modify or even override the germline‐determined HLA diversity.

Indeed, tumours dynamically reshape the HLA‐I landscape through somatic alterations such as loss of heterozygosity (LOH), copy‐number deletions, or epigenetic silencing.^16^ These events reduce HLA‐I diversity (somatic HED), narrowing the antigenic repertoire presented to T cells and enabling immune escape. Therefore, while germline HED represents the baseline antigen presentation potential, somatic HED reflects the residual functional capacity after tumour evolution. Existing biomarkers like neoantigen and TMB focus on antigen generation but ignore antigen presentation efficiency, creating a gap in our ability to explain why some patients with high neoantigen loads still fail to respond to PD‐1 blockade.

To bridge this gap, we introduce deltaHED, defined as the difference between germline HED (baseline potential) and somatic HED (post‐tumour evolution state). DeltaHED quantifies the extent of functional erosion in HLA‐I–mediated antigen presentation, integrating both host‐intrinsic potential and tumour‐acquired immune escape. This study aims to establish the prognostic value of deltaHED across multiple cancer types and explore its biological underpinnings through mutational and pathway analyses. To validate its universal utility, we assessed deltaHED in a multicohort framework encompassing refractory metastatic nasopharyngeal carcinoma (R/M NPC), melanoma, and esophageal squamous cell carcinoma (ESCC) patients treated with PD‐1 blockade as monotherapy or combined with chemotherapy. This design allows us to unravel conserved mechanisms of HLA‐mediated immune evasion across diverse clinical contexts.

## 2 RESULT

### 2.1 DeltaHED is an independent prognostic factor for PD‐1 blockade monotherapy in R/M NPC and melanoma

We analysed 164 patients with R/M NPC from the POLARIS‐02 trial (NCT02915432), all receiving toripalimab (anti‐PD‐1) monotherapy. Whole‐exome sequencing (WES) data were available for these patients (Table S1). DeltaHED, defined as germline HLA evolutionary divergence (HED) minus somatic HED, quantifies tumour‐mediated HLA‐I heterozygosity loss. Germline HED measures the evolutionary distance between inherited HLA‐I alleles, while somatic HED reflects post‐tumour evolution HLA diversity. Using maximally selected rank statistics, we stratified patients into high deltaHED (> 4.64, *n* = 15) and low deltaHED (≤4.64, *n* = 149) groups.

**FIGURE 1:**
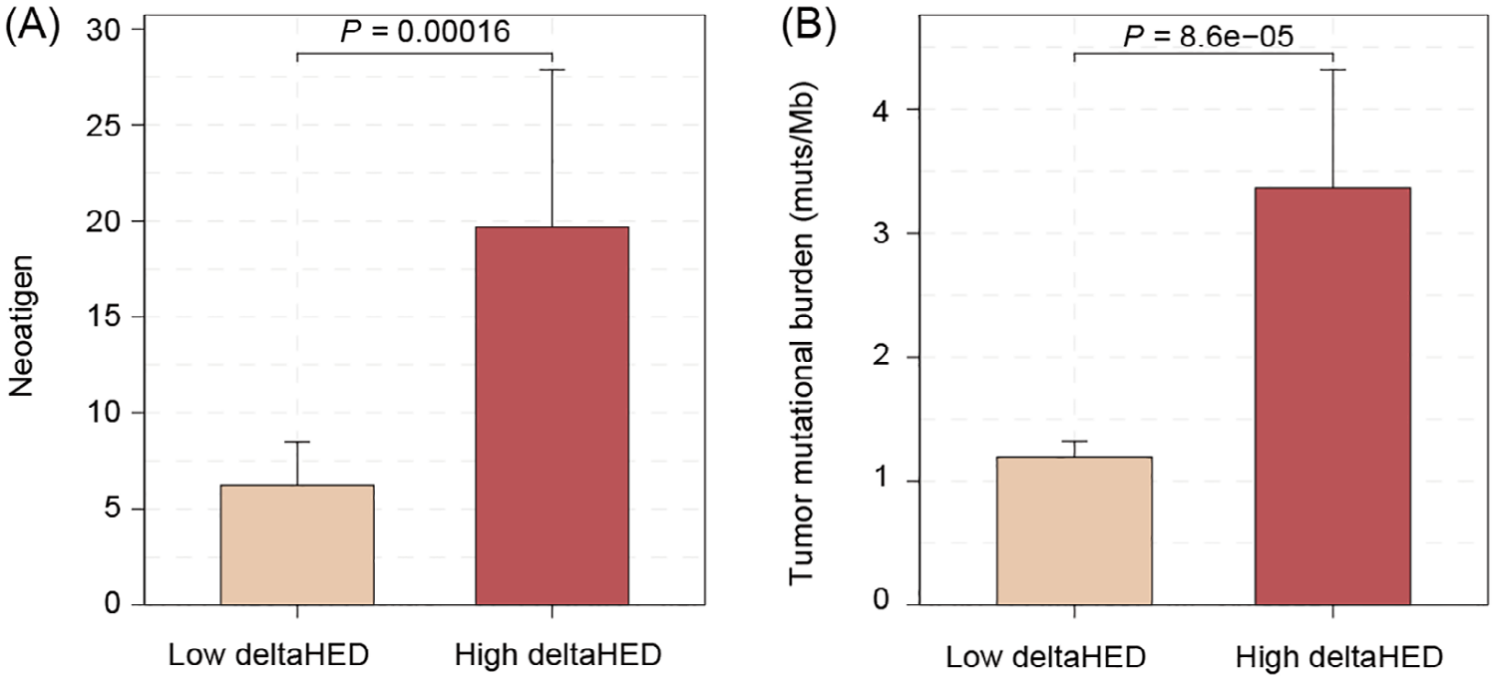
High deltaHED correlates with increased neoantigen burden and higher tumour mutational burden (TMB). (A) High deltaHED shows significantly reduced neoantigen burden compared to low deltaHED. (B) High deltaHED shows significantly lower TMB compared to low deltaHED.

High deltaHED tumours exhibited significantly higher neoantigen counts (median: 1.00 vs. .48, *p* < .001, Figure 1A) and tumour mutational burden (TMB; median: 2.5 vs. .9 mutations/Mb, *p* < .001, **Figure** **1B**) compared to low deltaHED tumours. Interestingly, higher neoantigens and TMB were observed in patients with LOH, yet neither germline HED nor somatic HED showed any correlation with neoantigens or TMB (Figure S1). These paradoxical findings suggest the need for further exploration of deltaHED's unique ability to capture dynamic HLA‐I erosion during tumour evolution.

**FIGURE 2:**
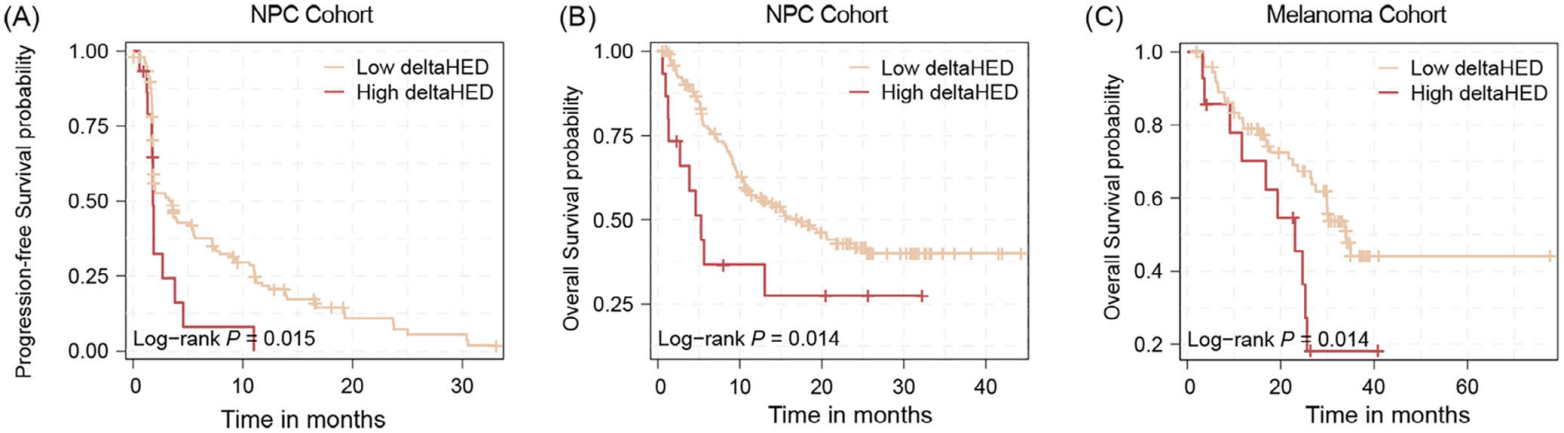
Survival analysis in different deltaHED group in refractory locally recurrent and/or metastatic nasopharyngeal carcinoma (R/M NPC) cohort and melanoma cohort. (A) The progression‐free survival (PFS) analysis in different deltaHED group in R/M NPC cohort. (B) The overall survival (OS) analysis in different deltaHED group in R/M NPC cohort. C is the OS analysis in melanoma group.

Despite higher neoantigen/TMB, patients in the high deltaHED group had a significantly worse PFS compared to those in the low deltaHED group (1.81 months, 95% CI [1.74–NA], vs. 3.45 months, 95% CI [1.87–5.42], *p* = .02, Figure 2A). Similarly, a significantly worse OS was observed in the high delta HED group (5.27 months, 95% CI [2.70–NA] vs. 17.00 months, 95% CI [11.30–NA], *p* = .02, Figure 2B). Multivariable Cox regression analysis demonstrated that deltaHED was an independent prognostic factor among TMB, PD‐L1, EBV DNA titre, or other clinical variables (for PFS: HR, 2.07; 95% CI [1.07–4.07]; *p* = .03; for OS: HR, 2.22; 95% CI [1.06–4.62]; *p* = .03, Tables 1 and 2). Post hoc power analysis based on observed event numbers and hazard ratios indicated approximately 83% power for PFS and 79% power for OS at α = .05. In contrast, germline HED, somatic HED, and somatic HLA‐I LOH showed no survival association (Figure S2). And neither PD‐L1 expression nor TMB alone was significantly associated with PFS or OS. However, combining TMB with deltaHED improved prognostic stratification, as patients with high TMB and high DeltaHED had the poorest outcomes (Figure S3).

**TABLE 1:** Univariate analysis and multivariate analysis of deltaHED in PFS.

Factors	Univariate analysis HR (95% CI)	*p* value	Multivariate analysis HR (95% CI)	*p* value
**Sex** (Female vs. male)	1.10 (.69–1.80)	.70	—	—
**Age**	1.00 (.99–1.00)	.48	—	—
**Weight**	.99 (.98–1.00)	.42	—	—
**ECOG** (0 vs. 1)	.98 (.68–1.40)	.91	—	—
**Liver metastasis** (yes vs. no)	1.50 (1.00–2.10)	.03	1.64 (.94–2.05)	.10
**Previous radiotherapy** (yes vs. no)	.59 (.32–1.10)	.09	—	—
**PD L1 status** ^a^ (negative vs. positive)	.86 (.58–1.30)	.46	—	—
**TMB** ^b^ (> 1.31 vs. ≤1.31)	.91 (.63–1.30)	.64	—	—
**Baseline EBV** (≥10 000 vs. < 10 000)	1.50 (1.00–2.10)	.04	1.45 (.90–1.97)	.15
**deltaHED** (> 4.64 vs. ≤4.64)	2.00 (1.10–3.70)	.02	2.17 (1.07–4.07)	.03

Abbreviations: ECOG, Eastern Cooperative Oncology Group; PD‐L1, programmed death‐ligand 1; TMB, tumour mutation burden.

^a^ Positive PD‐L1 expression is defined as ≥1% tumour cells expressing PD‐L1 by SP142 immunohistochemistry staining.

^b^ Mutations per megabase.

**TABLE 2:** Univariate analysis and multivariate analysis of delta HED in OS.

Factors	Univariate analysis HR (95% CI)	*p* value	Multivariate analysis HR (95% CI)	*p* value
**Sex** (female vs. male)	1.40 (.78–2.60)	.25	—	—
**Age**	1.00 (.99–1.00)	.58	—	—
**Weight**	.99 (.97–1.00)	.18	—	—
**ECOG** (0 vs. 1)	1.60 (.98–2.50)	.06	—	—
**Liver metastasis** (yes vs. no)	1.50 (.97–2.30)	.07	—	—
**Previous radiotherapy** (yes vs. no)	1.10 (.51–2.40)	.82	—	—
**PD L1 status** ^a^ (negative vs. positive)	.85 (.52–1.40)	.51	—	—
**TMB** ^b^ (> 1.31 vs. ≤1.31)	1.10 (.69–1.70)	.74	—	—
**Baseline EBV** (≥10 000 vs. < 10 000)	1.70 (1.10–2.70)	.02	1.69 (1.08–2.66)	.02
**deltaHED** (> 4.64 vs. ≤4.64)	2.20 (1.20–4.70)	.02	2.22(1.07–4.62)	.03

Abbreviations: ECOG, Eastern Cooperative Oncology Group; PD‐L1, programmed death‐ligand 1; TMB, tumour mutation burden.

^a^ Positive PD‐L1 expression is defined as ≥1% tumour cells expressing PD‐L1 by SP142 immunohistochemistry staining.

^b^ Mutations per megabase.

To further validate the prognostic value of deltaHED in other cancer types, a cohort with 88 melanoma patients treated with anti‐PD‐1 immunotherapy was used for validation.^17^, ^18^ Using a cut‐off value of 5.75 (calculated with maximally selected rank statistics), patients were stratified into two groups: the high deltaHED group (> 5.75, *n* = 14) and the low deltaHED group (≤5.75, *n* = 74). Patients in the high deltaHED group had a significantly worse median OS compared to those in the low deltaHED group (23.1 months, 95% CI [11.70–NA] vs. 34.00 months, 95% CI [29.90–NA], *p* = .01; Figure 2C). The Cox regression analysis shows HR of 2.41 (95% CI 1.17–4.98; *p* = .03), and post hoc power analysis indicated 75% power at α = .05.

### 2.2 DeltaHED is associated with prognosis for PD‐1 blockade plus chemotherapy in refractory ESCC

To assess whether deltaHED could serve as a prognostic indicator in patients receiving PD‐1 blockade with chemotherapy, we examined whole‐exome sequencing (WES) data from participants enrolled in the JUPITER‐06 phase III trial.^19^ 477 patients with advanced ESCC were stratified into four groups based on their treatment (chemotherapy combined with toripalimab [*n* = 235] or placebo [*n* = 242]) and deltaHED levels (high deltaHED > 2.33; low deltaHED ≤ 2.33).

**FIGURE 3:**
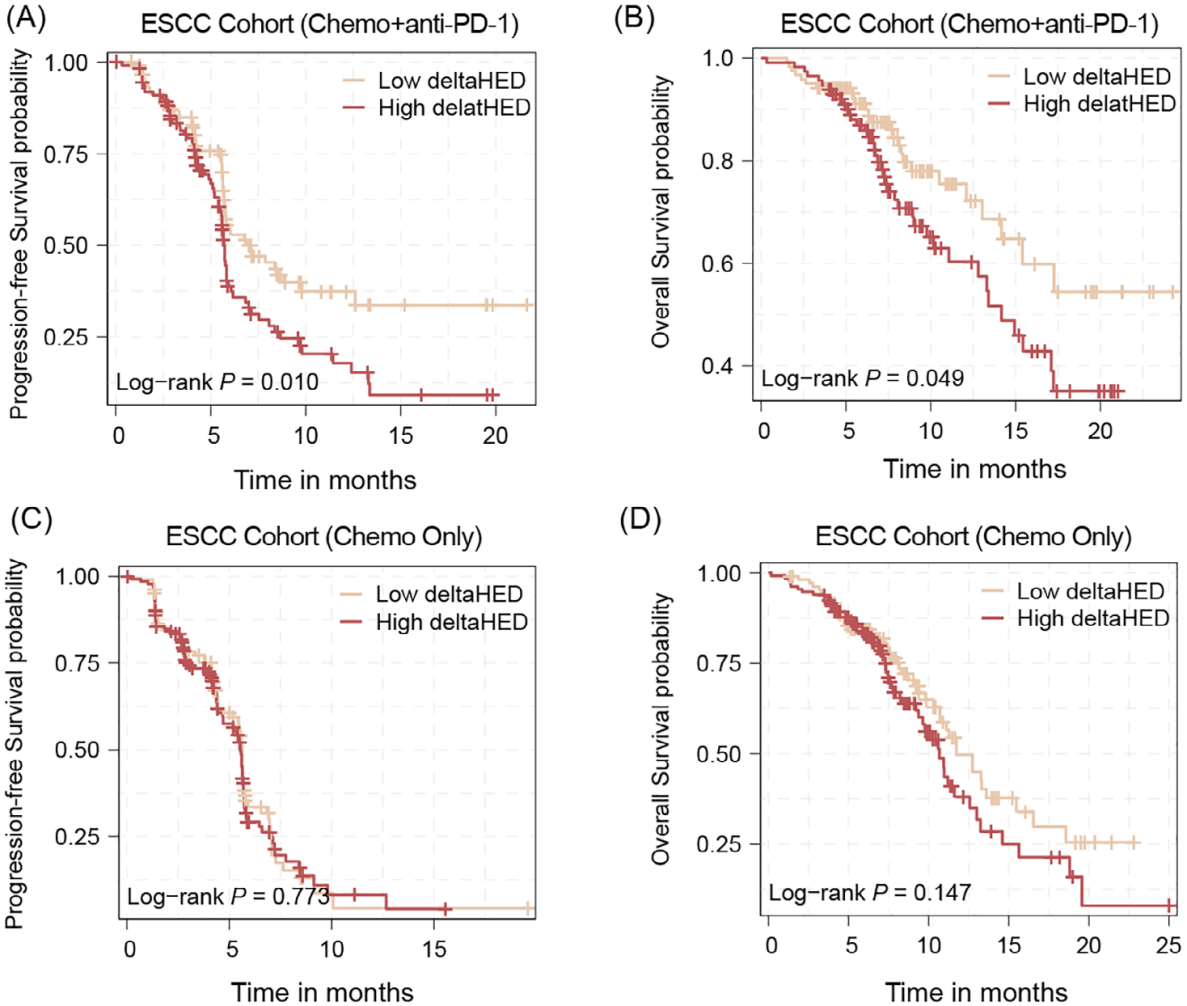
Survival analysis in different deltaHED group in esophageal squamous cell carcinoma (ESCC) receiving chemotherapy plus PD‐1 blockade or placebo. (A, B) The PFS analysis and OS in ESCC patients with chemotherapy plus PD‐1 blockade in high deltaHED group (*n* = 114) and low deltaHED group (*n* = 121). C and D are the PFS analysis and OS analysis in ESCC patients with chemotherapy plus placebo in high deltaHED group (*n* = 134) and low deltaHED group (*n* = 108).

In the toripalimab plus chemotherapy arm (*n* = 235), patients with high deltaHED (*n* = 114) had significantly shorter PFS (5.73 months, 95% CI 5.57–6.07) compared to those with low deltaHED (*n* = 121; PFS: 7.13 months, 95% CI 5.77–12.60; *p* < .01, Figure 3A). The achieved statistical power for this analysis was approximately 80% at α = .05, supporting the robustness of the observed association. Similarly, OS was also significantly reduced in the high deltaHED subgroup (14.2 months, 95% CI 11.1–NA) versus the low deltaHED subgroup (median OS not attained, 95% CI 15.4–NA; *p* = .049, Figure 3B), with an estimated power of 63%. Moreover, PD‐L1 expression and TMB alone were not significantly associated with survival (Figure S4A–D). When combined with deltaHED, patients with High TMB and High deltaHED showed significantly shorter PFS (*p* < .05), whereas the difference in OS was not significant (Figure S4E and F). However, in the placebo plus chemotherapy arm (*n* = 242), deltaHED stratification showed no prognostic significance (Figure 3C and D). It suggests the specific association with PD‐1 blockade prognosis rather than chemotherapy alone.

### 2.3 DeltaHED is associated with antigen presentation and processing pathway in R/M NPC and ESCC

As previously mentioned, a significantly higher level of neoantigen number and TMB was found in patients with high deltaHED compared with low deltaHED. Paradoxically, despite these immunogenomic features indicative of enhanced tumour immunogenicity, the high deltaHED subgroup had poor prognosis. This inverse association suggests that deltaHED may serve as a surrogate marker for HLA‐I‐mediated antigen presentation dysfunction, which could override the potential benefits of high neoantigen/TMB and drive immune evasion during PD‐1 blockade therapy.

**FIGURE 4:**
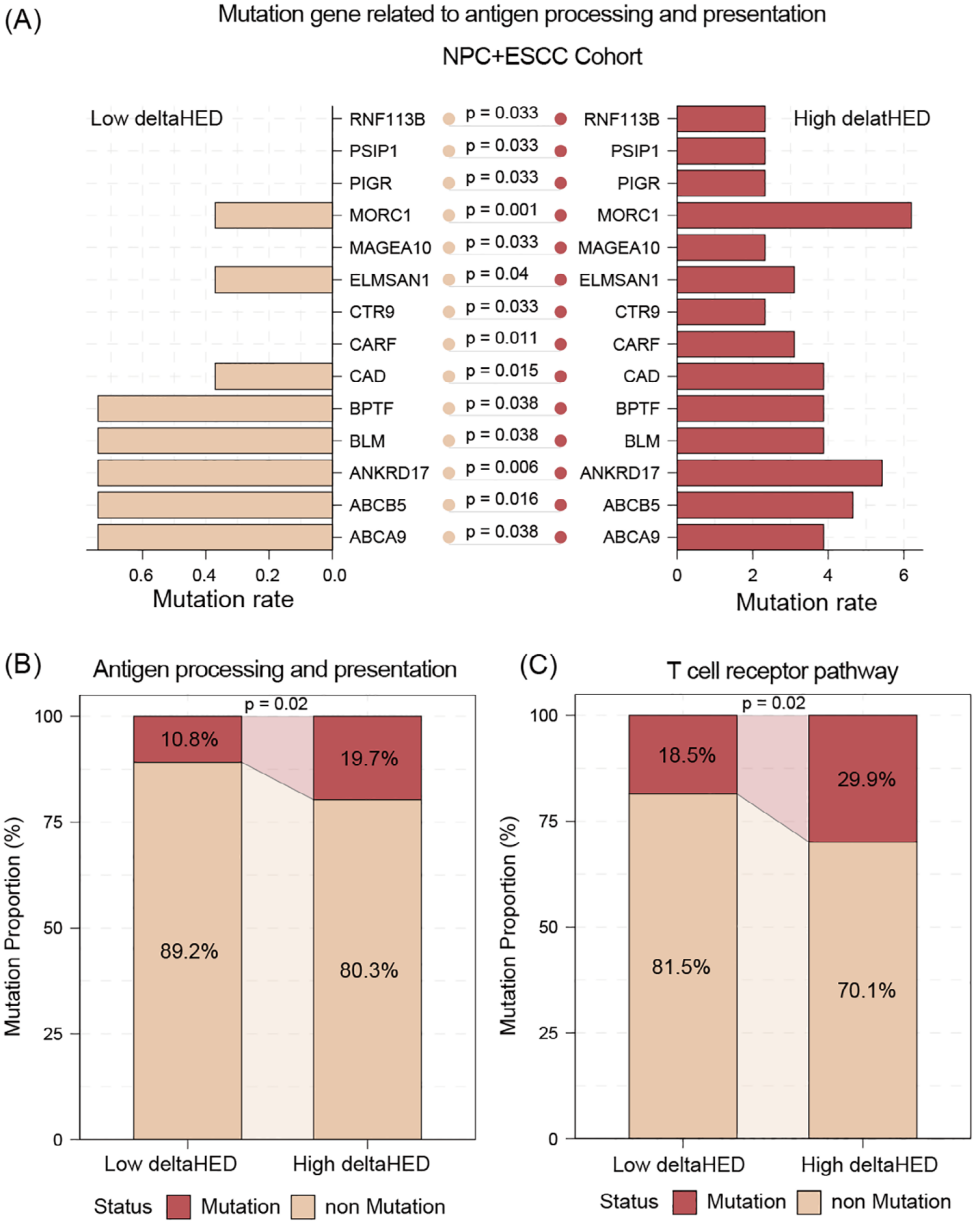
Mutation gene and pathway analysis in R/M NPC and ESCC cohort with low deltaHED (*n* = 259) and high deltaHED groups (*n* = 127). (A) The mutation portion of antigen processing and presentation genes. (B) The mutation portion of antigen processing and presentation pathway and T‐cell receptor pathway.

To investigate the mechanistic basis of this phenomenon, we performed comparative genomic analyses between high and low deltaHED subgroups across both R/M NPC and ESCC cohorts. The high deltaHED subgroup had significantly higher mutation frequencies in genes related to antigen processing and presentation (e.g., *RNF113B, MORC1, BLM, BPTF, ABCB5*; **Figure** **4A**). Furthermore, significantly higher rate of mutations in the antigen processing and presentation pathway (19.7% vs. 10.8%, *p* = .02) as well as T‐cell receptor signalling pathway (29.9% vs. 18.5%, *p* = .02) was observed in high deltaHED group (**Figure** **4B**). Consistent trends were observed when the NPC and ESCC cohorts were analysed separately (Figure S5), supporting that deltaHED‐associated pathway alterations are conserved across cancer types. These alterations likely impair HLA‐I‐dependent neoantigen presentation and disrupt T‐cell recognition, ultimately diminishing the efficacy of PD‐1 blockade despite elevated tumour immunogenicity.

## 3 DISCUSSION

Our study identifies deltaHED—a novel biomarker quantifying tumour‐mediated HLA‐I heterozygosity loss—as a robust prognostic factor for PD‐1 blockade therapy across multiple cancer types. While prior studies have linked germline HED and somatic LOH to immunotherapy outcomes in other malignancies,^20^, ^21^, ^22^ these associations were absent in R/M NPC. By integrating germline HED (reflecting inherited HLA diversity) and somatic HED (post‐tumour evolution HLA erosion), deltaHED uniquely captures the functional impairment of antigen presentation, addressing a critical gap left by conventional biomarkers like TMB and neoantigen load.

Consistent with earlier reports in NPC,^23^, ^24^ we observed that TMB and neoantigen levels failed to predict PD‐1 blockade efficacy, despite their established utility in other cancers. Paradoxically, high deltaHED tumours have higher TMB and neoantigen counts yet worse survival—a finding that underscores the limitations of focusing solely on antigen generation. Our data suggest that deltaHED reflects a deeper layer of immune dysfunction: the tumour's ability to present antigens, not just generate them. This aligns with the growing recognition that HLA‐I diversity loss and antigen presentation defects are key drivers of immune evasion.^25^, ^26^

The enrichment of mutations in antigen processing/presentation and TCR pathways in high deltaHED tumours provides mechanistic support for this hypothesis. These alterations create a “double hit” scenario: tumours evade immune detection by restricting antigen presentation and suppressing T‐cell recognition. This dual mechanism explains why high deltaHED patients, despite having immunogenic tumours (high TMB/neoantigens), derive limited benefit from PD‐1 blockade.

DeltaHED's specificity to PD‐1 blockade—not chemotherapy—was strikingly demonstrated in the ESCC cohort. Patients receiving toripalimab plus chemotherapy showed markedly worse outcomes with high deltaHED, whereas no association was observed in the chemotherapy‐alone arm. This underscores deltaHED's role as a biomarker of immune resistance, not general tumour aggressiveness. Importantly, deltaHED diverges from neoantigen, which only estimates potential antigens without accounting for presentation efficiency. Current neoantigen prediction algorithms are further limited by imperfect HLA‐peptide binding affinity models,^27^ whereas deltaHED directly measures HLA‐I functional diversity, offering a more reliable readout of antigen presentation capacity.

The HLA region exhibits extensive genetic diversity across populations, with allele and haplotype frequencies shaped by geographic and evolutionary pressures.^28^ Our ESCC and NPC cohorts were composed primarily of East Asian patients, whereas the melanoma cohort included European and American populations. The consistent prognostic performance of deltaHED across these ethnic distinct groups suggests that this biomarker may capture a universal feature of tumour and immune interaction rather than population‐specific HLA patterns. Nevertheless, larger studies across ancestrally diverse and genetically admixed cohorts will be needed to further confirm its generalisability and clinical utility.

While our findings are validated across three independent cohorts, several limitations warrant consideration. The use of clinical trial cohorts (POLARIS‐02, JUPITER‐06) may limit generalisability to real‐world populations. However, the inclusion of diverse cancer types (NPC, melanoma, ESCC) strengthens external validity. Although pathway analyses suggest antigen presentation defects, functional studies are needed to confirm causality. The optimal deltaHED cut‐off values varied among cancer types, reflecting cohort size, population‐specific HLA allele distributions, and tumour‐type‐specific immunogenomic features rather than methodological differences. These thresholds were determined using maximally selected rank statistics, which define data‐driven survival‐based cut‐offs within each cohort.^29^ As deltaHED is derived from exome data, it captures HLA‐I diversity loss but not its molecular causes. Future multi‐omics analyses integrating copy number, methylation, and expression profiles will be needed to clarify these mechanisms.

In conclusion, our findings reveal a strong clinical and genomic association between high deltaHED and poor outcomes, but functional experiments are required to confirm a direct causal role in impaired antigen presentation and T‐cell recognition.

## 4 METHODS

### 4.1 Patients and data collection

Details regarding the design and outcomes of the POLARIS‐02 (Phase II) and JUPITER‐06 (Phase III) trials have been reported in prior publications.^19^, ^24^ This study was approved by the ethics committee of Sun Yat‐sen University Cancer Center and performed in accordance with the Declaration of Helsinki. Written informed consent was obtained from all study participants. This study was conducted in alignment with the Transparent Reporting of a Multivariable Prediction Model for Individual Prognosis or Diagnosis (TRIPOD) reporting guidelines.

### 4.2 Whole‐exome sequencing and genomic analysis

Whole‐exome sequencing (WES) was performed using the SureSelect Human All Exon V6 capture kit (Agilent Technologies, Santa Clara, CA) on paired tumour and corresponding blood samples. Genomic aberrations, including microsatellite stability, nucleotide variations, short and long insertions and deletions (indels), copy number alterations, gene rearrangements, and fusions, were evaluated. BWA‐MEM was used for read alignment, Picard for data processing, and GATK for variant calling and filtering in accordance with the Broad Institute's Best Practices.

Tumour mutational burden (TMB) was determined by analysing somatic mutations encompassing coding base substitutions and indels, expressed as mutations per megabase (Mb). For neoantigen prediction, the 4‐digit HLA type for each sample was inferred using Polysolver. Putative neoantigens were predicted for each patient by defining all novel amino acid 9‐mers and 10‐mers resulting from each somatic nonsynonymous point mutation. The predicted binding rank, which serves as a proxy for the predicted binding affinity to the patient's germline HLA alleles, was used to evaluate potential neoantigens. Strong binders were defined as those with a rank of < .5%, while weak binders had ranks between .5% and 2%, as determined using NetMHCpan (v3.0).

### 4.3 Computational identification of HLA‐I‐restricted neopeptides

Each non‐synonymous SNV or short insertion/deletion was converted into a 17–amino acid peptide centred on the mutated site. Adjacent SNVs were adjusted using MAC52. The 17‐mer sequence was then segmented into 11‐mer sequences via a sliding window technique, which was employed to predict HLA‐I‐binding for neopeptides with the use of Net MHC pan‐4.0.^30^ We retained all peptides with the binding affinity of more than 500, and the control group is less than 500 for further investigation.

### 4.4 Calculation of HED

HED was computed following the approach described by Pierini and Lenz.^11^ Briefly, protein sequences of exons 2 and 3, corresponding to the peptide‐binding domains, were extracted from each allele of each patient's HLA‐I genotype. Protein sequences were derived from the ImMunoGeneTics/HLA database, and exons for the variable peptide‐binding domains were selected based on annotations from the Ensembl database. The Grantham distance metric was used to quantify divergences between allele sequences, accounting for the physiochemical properties of amino acids, including composition, polarity, and volume, to reflect functional similarity between sequences.^30^ For each HLA‐I locus containing two alleles, peptide‐binding domain sequences were aligned, and the evolutionary distance was derived from cumulative differences in amino acid composition, polarity, and volume.^31^, ^32^

Ambiguous HLA genotypes were resolved using representative G‐group alleles based on the IMGT/HLA database (release 3.41.0). Homozygous loci were assigned a Grantham distance of 0, indicating no divergence. Protein sequence alignment was performed using Clustal Omega (EMBL‐EBI) with default parameters. Insertions and deletions (indels) were handled according to Clustal's default treatment and excluded from distance calculations. The final HED score per patient was calculated as the average of Grantham distances across the HLA‐A, ‐B, and ‐C loci.

### 4.5 Calculation of deltaHED

We employed the LOHHLA algorithm to determine whether there was a LOH in HLA‐I. In cases where LOH was identified in HLA, we proceeded to eliminate the lost HLA alleles, preserving only the unlost alleles. For example, given HLA alleles A02:06, A33:03, B51:01, B40:01, C14:02, and C02:02, if B51:01 was determined to be lost, we would remove it, retaining the remaining alleles, that is, A02:06, A33:03, B40:01 (homozygous), C14:02, and C02:02. Following this, we recalculated the HED, defined now as the somatic HED. Finally, we calculated deltaHED, which equals the difference between the germline HED and the somatic HED. The optimal deltaHED cut‐off values for each cancer type (NPC, melanoma, and ESCC) were determined using *maximally selected rank statistics*, which identify the threshold that best separates patients with different survival outcomes based on deltaHED distribution within each cohort.

### 4.6 Analysis of genetic mutation frequencies and KEGG pathway alterations

To compare genetic mutation frequencies and related pathways between the low deltaHED and high deltaHED groups, somatic mutation data were converted into Mutation Annotation Format (MAF) files. Mutation frequencies for individual genes were calculated for each group. Pathway‐level alterations were assessed using the Kyoto Encyclopedia of Genes and Genomes (KEGG) database. A pathway was considered altered in a patient if one or more genes within that pathway harboured somatic mutations. Pathways of interest included antigen processing and presentation, T‐cell receptor signalling, and other immune‐related pathways. Statistical comparisons of mutation frequencies and pathway alterations between the low and high deltaHED groups were performed using Fisher's exact test or chi‐square test, as appropriate.

### 4.7 Statistical analyses

Tumour response was assessed independently according to the RECIST version 1.1 criteria. PFS was quantified as the duration from the initial treatment to the first recorded occurrence of disease progression or death, whichever transpired first. OS was defined as the time between the initial dose of toripalimab and death resulting from any cause. Patients experiencing progression identified in the second or subsequent imaging were regarded as having secondary progression.

All statistical analyses were carried out using R software (v3.6.1; R Foundation for Statistical Computing, Vienna, Austria). A two‐tailed *p*‐value of less than .05 was defined as statistical significance for power testing. The neoantigen number in patients with different deltaHED, germline HED, somatic HED and TMB was compared using the Mann–Whitney test. Kaplan–Meier survival curves were generated, and differences between groups were evaluated using the log‐rank test. HR 95% CI were calculated using the Cox proportional hazards regression model.

## AUTHOR CONTRIBUTIONS

Fenghua Wang, Xiaoli Wei, Fei Han, and Jianying Xu conceived and designed the study. Fenghua Wang, Xiaoli Wei, and Jianying Xu collected and assembled the data. Jianying Xu and Jicheng Yao performed data analysis and interpretation. All authors contributed to manuscript writing and approved the final version.

## CONFLICT OF INTEREST STATEMENT

All other authors declare no competing interests.

## ETHICS STATEMENT

This study was approved by the institutional ethics committee of Sun Yat‐sen University Cancer Center (Approval Number: NCT02915432) and conducted in accordance with the principles of the Declaration of Helsinki. Written informed consent was obtained from all participants prior to their inclusion in the study.

## Supporting information



Supporting Information

Supporting Information

Supporting Information

Figure S4: Comparison of deltaHED with PD‐L1 and TMB in the advanced ESCC cohort. (A–D) Kaplan–Meier analyses of PFS and OS according to PD‐L1 expression and TMB in patients with ESCC receiving PD‐1 blockade plus chemotherapy. PD‐L1 and TMB alone were not significantly associated with survival (Log‐rank *p* > .05). (E, F) When TMB was combined with deltaHED, patients with high TMB and high deltaHED showed significantly shorter PFS, while no significant difference was observed in OS.

Figure S5: Comparison of mutation frequencies in immune‐related pathways according to deltaHED level in R/M NPC and ESCC cohorts. Stacked bar plots show the proportion of tumours harbouring mutations in antigen processing and presentation and T‐cell receptor (TCR) signalling pathways, separately analysed for NPC (A, B) and ESCC (C, D). High deltaHED tumours exhibited higher mutation frequencies in these pathways across both cancer types.

Supporting Information

## Data Availability

The datasets generated and/or analysed during the current study are available from the corresponding author upon reasonable request. The raw sequencing data are not publicly available due to privacy and ethical restrictions but can be accessed through a data transfer agreement.
