# Supplementary material for: deltaHED predicts survival and immune evasion in PD‐1 blockade therapy: A multi‐cohort study across three cancer types
Source: Clin Transl Med. 2026 Jan 28;16(2):e70595. doi: 10.1002/ctm2.70595 (PMC12848521; doi:10.1002/ctm2.70595)
Supplement: Supplementary file 1 — Supporting Information [file CTM2-16-e70595-s003.docx]

| **Characteristics** | **No. of Patients (%)** |
| --- | --- |
| **Sex (%)** |  |
| Female | 27 ( 16.5) |
| Male | 137 ( 83.5) |
| **Age, years, median (range)** | 46 (22-71) |
| **Weight, kg, median (range)** | 60 (31.6-101.0) |
| **ECOG at Baseline (%)** |  |
| 0 | 56 ( 34.1) |
| 1 | 108 ( 65.9) |
| **Previous Radiotherapy (%)** |  |
| Yes | 148 ( 90.2) |
| No | 16 (9.8) |
| **Previous Chemotherapy (%)** | 164 (100.0) |
| **Histology** ^a^ |  |
| Keratinizing | 6 (3.7) |
| Nonkeratinizing | 158(96.3) |
| **Liver Metastasis (%)** |  |
| Yes | 88 ( 53.7) |
| No | 76 ( 46.3) |
| **PD L1 status (%)** ^b^ |  |
| Negative | 115 ( 70.1) |
| Positive | 46 ( 28.0) |
| NA | 5 (1.9) |
| **TMB (%)** ^c^ |  |
| >1.31 | 55 ( 33.5) |
| ≤1.31 | 109 ( 66.5) |
| **Baseline EBV DNA titer (%)** |  |
| ≥10,000 IU/mL | 90 ( 54.9) |
| <10,000 IU/mL | 65 (39.6) |
| NA | 9 (5.5) |
| **MSI status** |  |
| MSS | 163 (99.3) |
| MSI-H | 1 (0.7) |
| **deltaHED (%)** |  |
| >4.64 | 15 ( 9.1) |
| ≤4.64 | 149 ( 90.9) |

**Supplementary Table 1. Patient demographics and clinical characteristics** Abbreviations: EBV, Epstein-Barr virus; ECOG, Eastern Cooperative Oncology Group; N/A, not available; TMB: Tumor Mutation Burden; PD-L1, programmed death ligand-1; MSI: Microsatellite Instability; MSS: Microsatellite Stability; MSI-H: Microsatellite Instability High; ^a^ Classified according to American Joint Committee on Cancer and Union for International Cancer Control version 8th TNM staging system. ^b^ Positive PD-L1 expression is defined as ≥1% tumor cells expressing PD-L1 by SP142 immunohistochemistry staining. ^c^ mutations per megabase

**Supplementary Table 2. Association between deltaHED group and baseline characteristics**

| **Characteristics** | **High deltaHED** | **Low deltaHED** | ***P* value** |
| --- | --- | --- | --- |
| **Sex (%)** |  |  | 0.14 |
| Female | 1 | 26 |  |
| Male | 14 | 123 |  |
| **Age, years (%)** |  |  | 0.79 |
| >55 | 4 | 30 |  |
| ≤55 | 11 | 119 |  |
| **Weight, kg (%)** |  |  | 0.79 |
| >60 | 8 | 76 |  |
| ≤60 | 7 | 73 |  |
| **ECOG.at.Baseline (%)** |  |  | 1.00 |
| 0 | 5 | 51 |  |
| 1 | 10 | 98 |  |
| **Previous.Radiotherapy (%)** |  |  | 0.06 |
| Yes | 12 | 136 |  |
| No | 3 | 13 |  |
| **Liver.Metastasis (%)** |  |  | 0.80 |
| Yes | 9 | 79 |  |
| No | 6 | 70 |  |
| **PD L1 status (%)** |  |  | 0.13 |
| Negative | 8 | 107 |  |
| Positive | 7 | 39 |  |
| **TMB (%)** |  |  | 0.001 |
| >1.31 | 13 | 65 |  |
| ≤1.31 | 2 | 84 |  |
| **Baseline EBV DNA titer (%)** |  |  | 0.74 |
| ≥10,000 IU/mL | 8 | 82 |  |
| <10,000 IU/mL | 4 | 61 |  |

Abbreviations: EBV, Epstein-Barr virus; ECOG, Eastern Cooperative Oncology Group; N/A, not available; TMB: Tumor Mutation Burden; PD-L1, programmed death ligand-1; ^b^ Positive PD-L1 expression is defined as ≥1% tumor cells expressing PD-L1 by SP142 immunohistochemistry staining. ^c^ mutations per megabase
