# Supplementary figures and images for: deltaHED predicts survival and immune evasion in PD‐1 blockade therapy: A multi‐cohort study across three cancer types
Source: Clin Transl Med. 2026 Jan 28;16(2):e70595. doi: 10.1002/ctm2.70595 (PMC12848521; doi:10.1002/ctm2.70595)

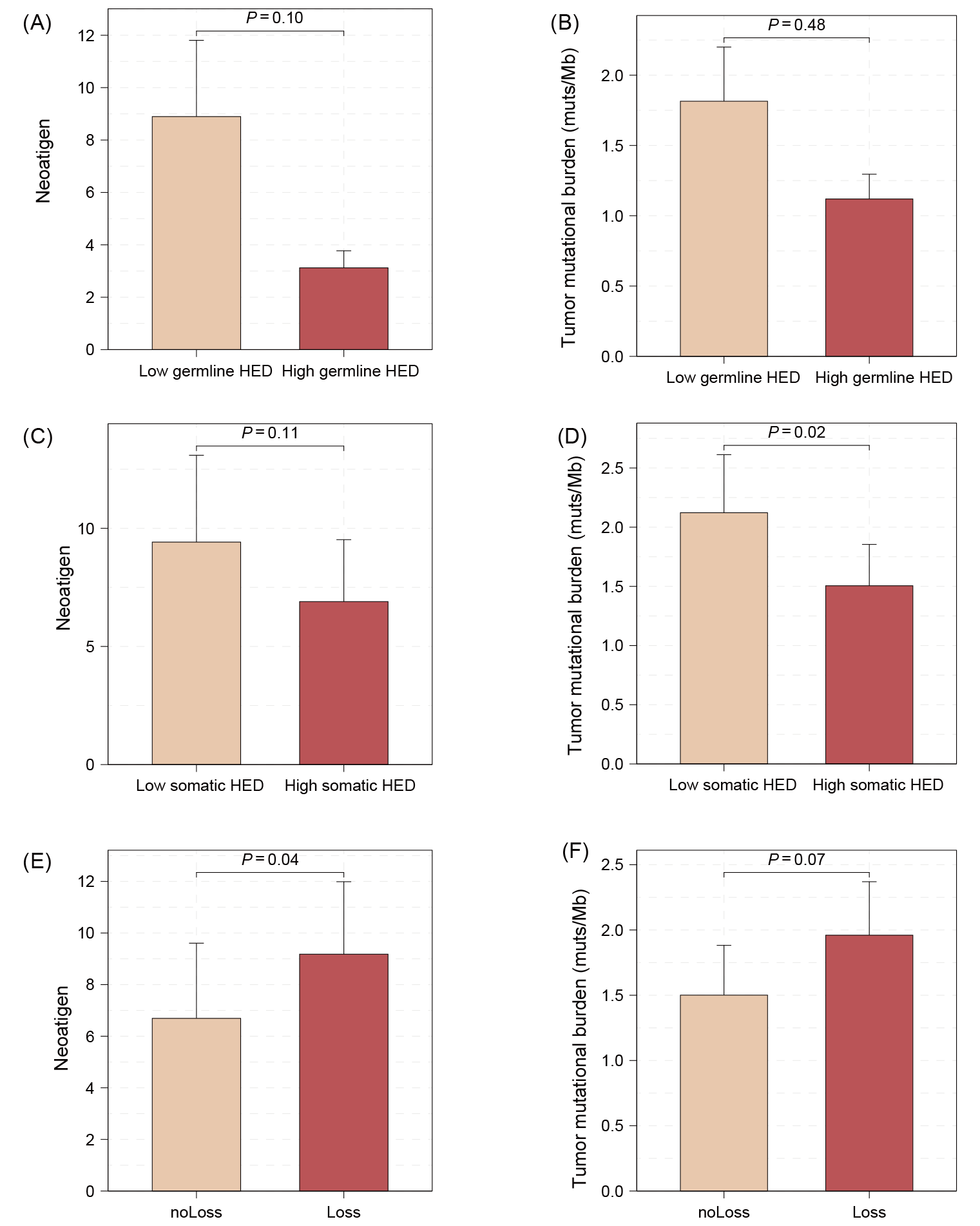

Supplement: Supplementary file 2 — Supporting Information [file CTM2-16-e70595-s006.tif]

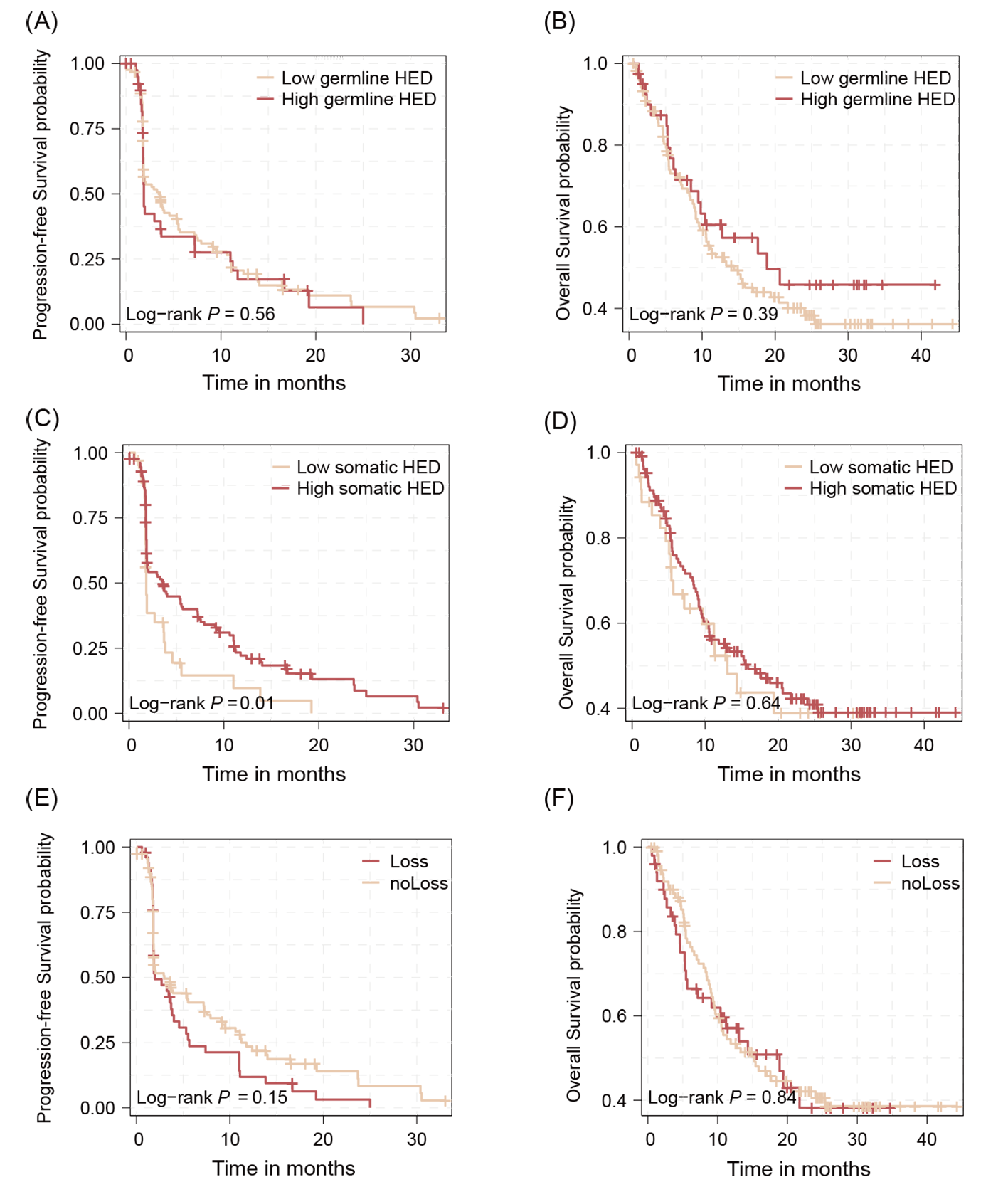

Supplement: Supplementary file 3 — Supporting Information [file CTM2-16-e70595-s004.tif]

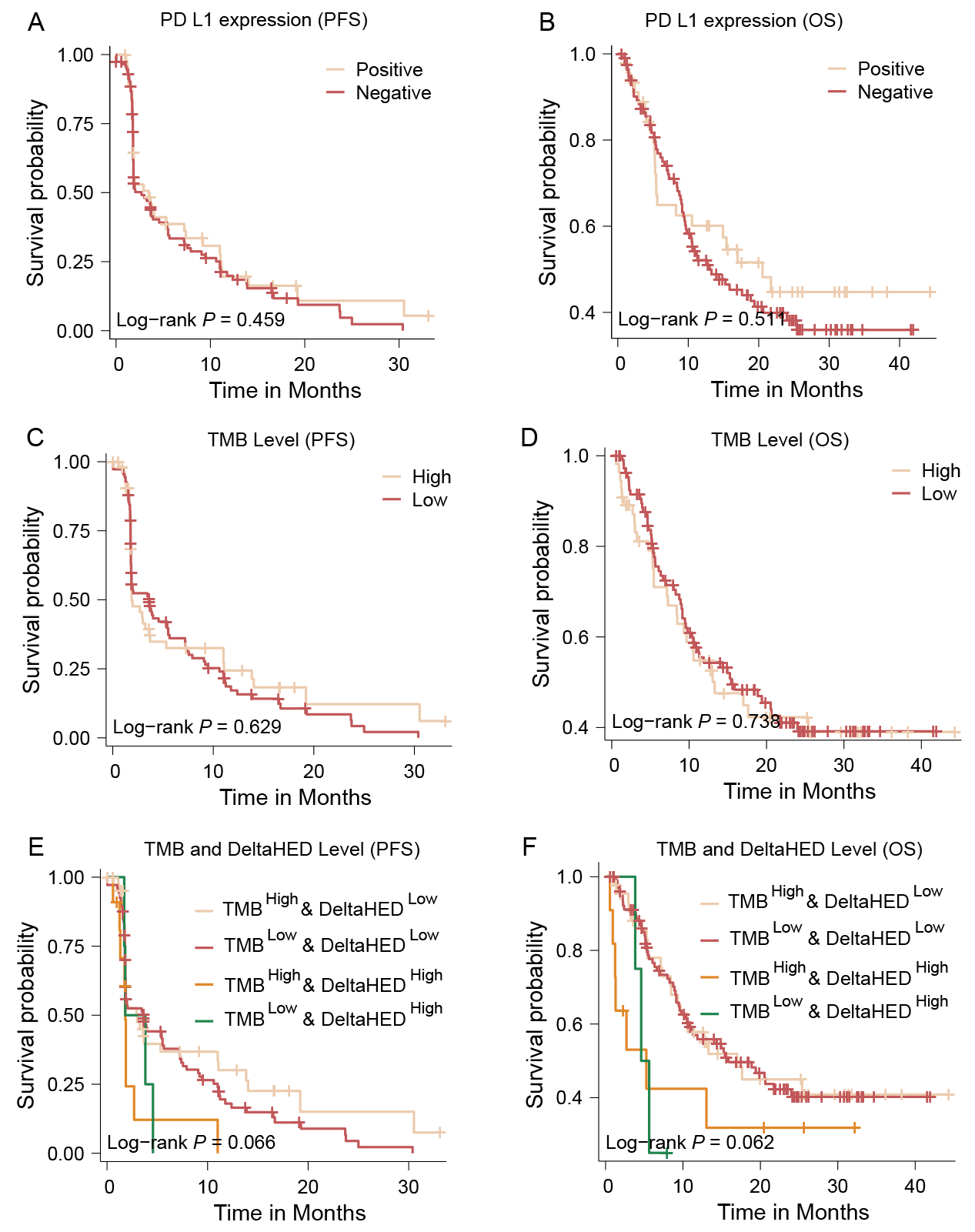

Supplement: Supplementary file 4 — Figure S4: Comparison of deltaHED with PD‐L1 and TMB in the advanced ESCC cohort. (A–D) Kaplan–Meier analyses of PFS and OS according to PD‐L1 expression and TMB in patients with ESCC receiving PD‐1 blockade plus chemotherapy. PD‐L1 and TMB alone were not significantly associated with survival (Log‐rank p > .05). (E, F) When TMB was combined with deltaHED, patients with high TMB and high deltaHED showed significantly shorter PFS, while no significant difference was observed in OS. [file CTM2-16-e70595-s001.tif]

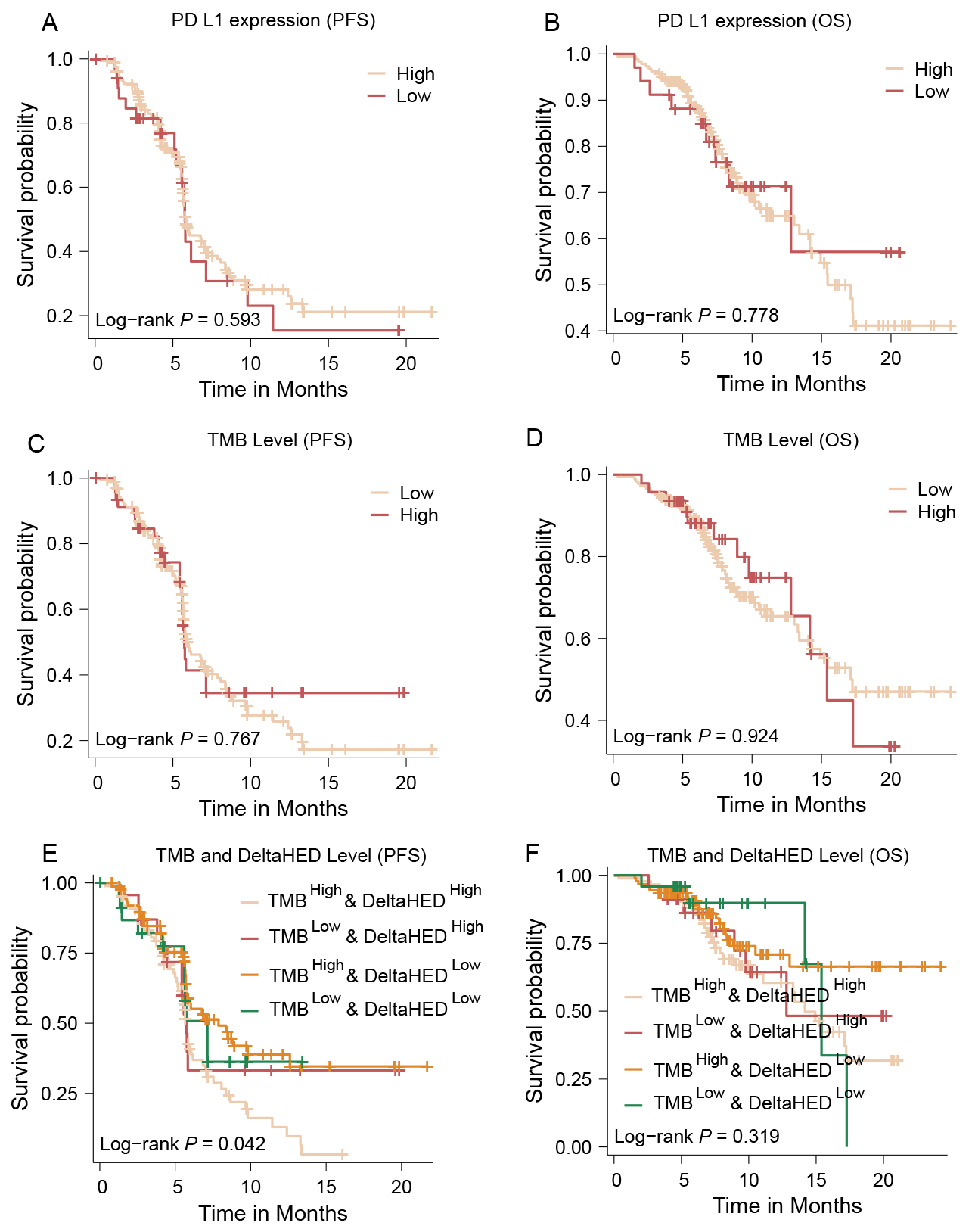

Supplement: Supplementary file 5 — Figure S5: Comparison of mutation frequencies in immune‐related pathways according to deltaHED level in R/M NPC and ESCC cohorts. Stacked bar plots show the proportion of tumours harbouring mutations in antigen processing and presentation and T‐cell receptor (TCR) signalling pathways, separately analysed for NPC (A, B) and ESCC (C, D). High deltaHED tumours exhibited higher mutation frequencies in these pathways across both cancer types. [file CTM2-16-e70595-s002.tif]

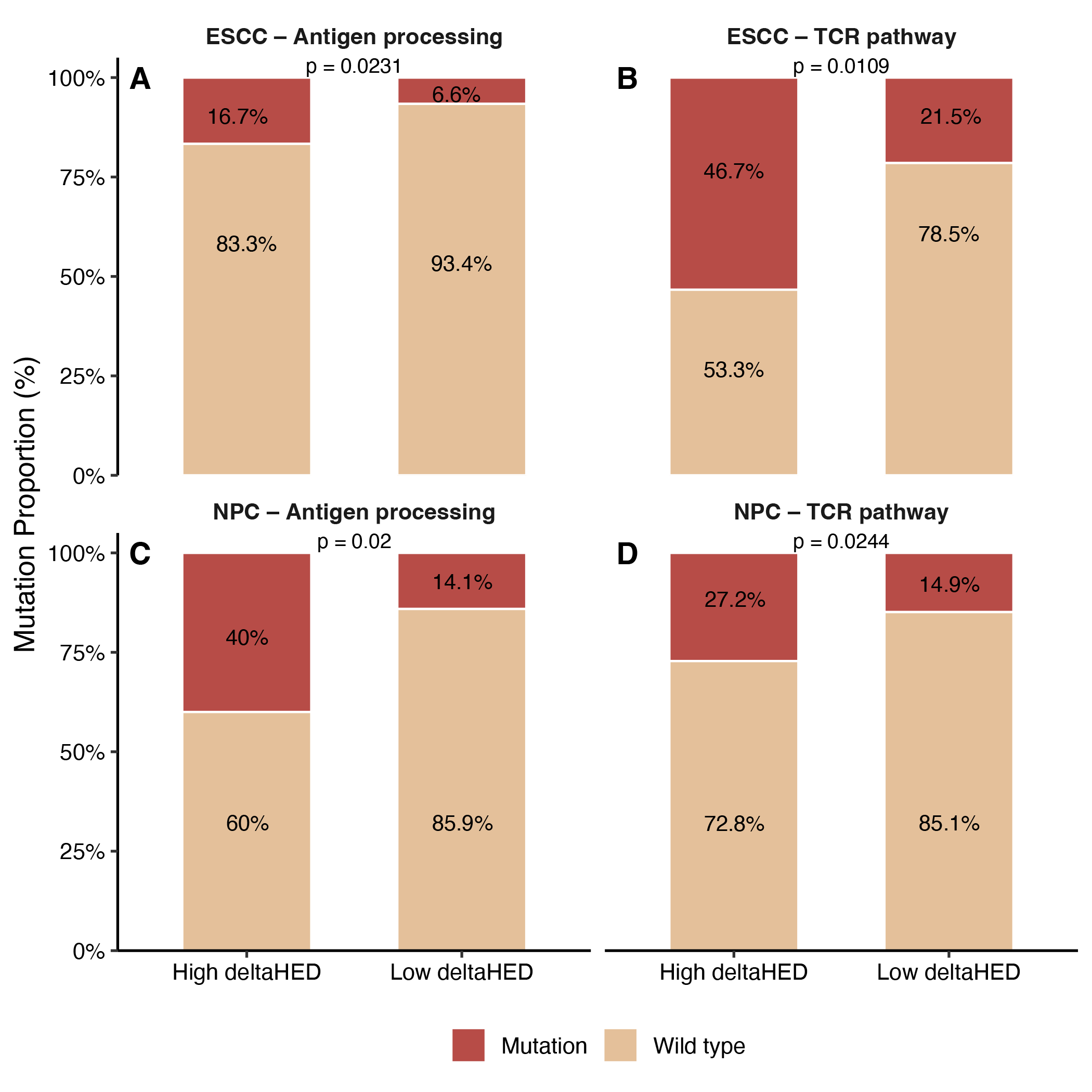

Supplement: Supplementary file 6 — Supporting Information [file CTM2-16-e70595-s005.tif]
